# The Feasibility of Percutaneous Dilatational Tracheostomy in Immunosuppressed ICU Patients with or without Thrombocytopenia

**DOI:** 10.1155/2022/5356413

**Published:** 2022-05-26

**Authors:** Marianne Angelberger, Michaela Barnikel, Alessia Fraccaroli, Johanna Tischer, Sofía Antón, Alexandra Pawlikowski, Mark op den Winkel, Hans Joachim Stemmler, Stephanie-Susanne Stecher

**Affiliations:** ^1^Department of Medicine II, University Hospital, LMU Munich, Munich 81377, Germany; ^2^Department of Medicine V, University Hospital, LMU Munich, Munich 81377, Germany; ^3^Department of Medicine III, University Hospital, LMU Munich, Munich 81377, Germany

## Abstract

**Background:**

Percutaneous dilatational tracheostomy (PDT) has become the preferred method in several intensive care units (ICUs), but data on PDT performed in immunosuppressed and thrombocytopenic patients are scarce. This study aimed to analyze the feasibility of PDT in immunosuppressed and thrombocytopenic patients compared to conventional open surgical tracheostomy (OST).

**Methods:**

We retrospectively analyzed the charts of patients who underwent PDT or OST between May 2017 and November 2020. Our outcomes were stoma site infections and bleeding complications.

**Results:**

63 patients underwent PDT, and 21 patients underwent OST. Distribution of gender ratio, age, SAPS II, time of ventilation before tracheostomy, and preexisting hematooncological diseases was comparable between the two groups. After allogeneic stem cell transplantation (alloSCT), patients were more likely to undergo PDT than OST (*p*=0.033). The PDT cohort suffered from mucositis more frequently (*p*=0.043). There were no significant differences in leucocyte or platelet count on the tracheostomy day. Patients with coagulation disorders and patients under immunosuppression were distributed equally among both groups. Stoma site infection was documented in five cases in PDT and eight cases in the OST group. Moderate infections were remarkably increased in the OST group. Smears were positive in six cases in the PDT group; none of these patients had local infection signs. In the OST group, smears were positive in four cases; all had signs of a stroma site infection. Postprocedural bleedings occurred in eight cases (9.5%) and were observed significantly more often in the OST group (*p*=0.001), leading to emergency surgery in one case of the OST group.

**Conclusion:**

PDT is a feasible and safe procedure in a predominantly immunosuppressed and thrombocytopenic patient cohort without an increased risk for stoma site infections or bleeding complications.

## 1. Introduction

Tracheostomies are among the most frequently performed procedures in mechanically ventilated intensive care patients [1, 2]. Percutaneous dilatational tracheostomy (PDT) and open surgical tracheostomy (OST) are the two methods used in intensive care units (ICUs). PDT has become the preferred method at several ICUs in the last few years. Prolonged mechanical ventilation and difficult weaning are the indications for PDT and do not differ from indications for conventional tracheostomy. Furthermore, oral intubation-associated complications such as laryngeal injury, tracheal stenosis, recurrent laryngeal nerve injury, formation of a tracheoesophageal fistula, and postextubation dysphagia [3, 4] are reasons that tracheostomy is the preferred way of weaning [2, 5].

One advantage of PDT is that it can be performed as a bedside procedure in critically ill patients. Furthermore, the technique itself leads to several advantages. PDT uses a single puncture and dilatation of the trachea leading to less traumatic injury to the surrounding tissue. In addition, the tracheal cannula has a close fitting to its stoma. Also, the way the tracheostomy is reversed differs significantly. While after PDT, the cannula must be removed, and tracheostomy closes itself, and OST requires a second surgery to close tracheostomy. The recommendation to perform PDT over OST is based on two meta-analyses [6, 7], showing moderate to slight evidence for a positive influence of PDT because PDT can be performed faster while no relevant disadvantages are seen.

The proportion of patients in ICU under immunosuppression due to hematological malignancies, chemotherapy-associated complications, and allogeneic stem cell (alloSCT) or solid organ transplantation increased over the last years. These patients suffer from quite similar problems regarding weaning, needing tracheostomies, in particular, to avoid complications and make weaning comfortable. Furthermore, in recent times, COVID-19 patients admitted to the ICU showed similar features [8]. Apart from respiratory emergencies, in patients under 18 years of age, the need for a continuous tracheostomy, the inability to extend the neck, morbid obesity, previous tracheostomy, and severe thrombocytopenia have been described as a contraindication for PDT [9, 10]. However, no data are published, especially in the setting of combined thrombocytopenia and mucositis. So far, there are also only small data available regarding infectious complications after PDT, especially in an immunosuppressed patient cohort [11, 12].

This study aimed to analyze the feasibility of PDT compared to OST in immunosuppressed and thrombocytopenic patients suffering predominantly from hematooncological diseases.

## 2. Materials and Methods

We retrospectively analyzed the feasibility and safety of PDT in an immunosuppressed and thrombocytopenic patient cohort at the Medical Intensive Care Unit, University Hospital of the Ludwig-Maximilians University, Munich, between May 2017 and November 2020.

The study was conducted following the amended Declaration of Helsinki [13]. The Ethics Committee of the Ludwig-Maximilians University Munich approved the protocol (IBD 18-598) and waived the need for informed consent because of the noninterventional and retrospective design of the investigation.

PDT was done for adult patients whenever there were no contraindications such as infections, short neck, or extreme obesity [2]. All PDTs were planned procedures.

This study predominantly included patients under immunosuppression caused by hematooncological diseases or following allogeneic stem cell or solid organ transplantation, to a small degree caused by autoimmune disorders, and patients with COVID-19. Smears were performed regularly every week or whenever clinically infection was suspected. To measure the physiological condition of patients in ICU, we developed the SAPS II score (simplified acute physiology score) [14] on the day when PDT was performed.

### 2.1. The Standard Procedure of PDT

**Figure 1:**
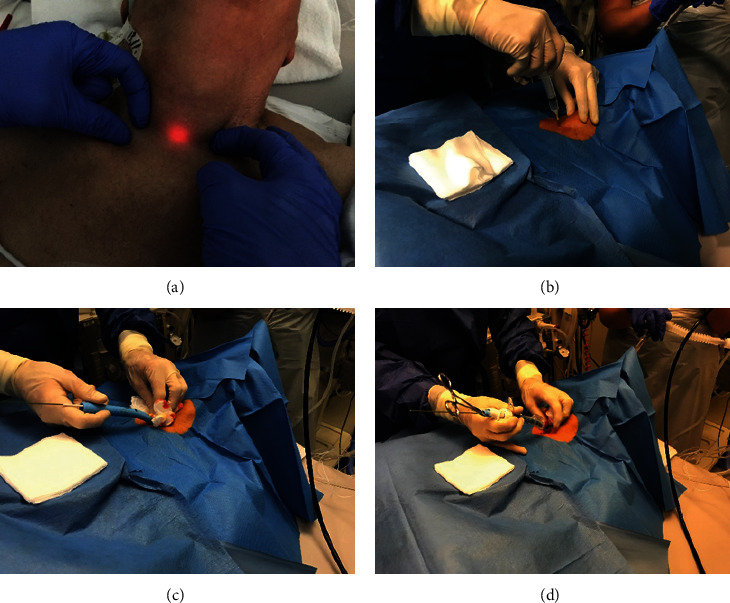
Stages of percutaneous dilatational tracheostomy. Diaphanoscopy (a), needle insertion midline, beneath second or third cartilage (b), dilatation with the Blue Rhino® (c), and insertion of the cannula (d).

In our ICU, the need for PDT is evaluated after about seven days of mechanical ventilation and unforeseeable extubation. An intensivist performs PDT with bronchoscopic guidance by another intensivist. Before PDT, the anatomic landmarks are palpated, and an ultrasound is performed to evaluate possible contraindications for PDT, such as a large isthmus of the thyroid gland or blood vessels in the operative area. The method was developed and first described by Ciaglia [15]; variations and further improvements led to several modifications, including the Ciaglia Blue Rhino® [16, 17]. Comparative studies show that this procedure carries the lowest risk for patients [18–20] regarding safety, success, and potential complications; at our center, we carry it out using the Ciaglia Blue Rhino® percutaneous tracheostomy introducer set (Cook Medical, USA). First, oral hygiene is done with an antiseptic. Next, the patient is positioned with hyperextension of the neck by placing a roll under the chest or shoulder. Under bronchoscopic guidance, the tube is pulled back into the larynx to find a suitable puncture site by diaphanoscopy (Figure 1(a)). Next, sterile draping is applied; the tracheostomy tube and the dilatator are prepared with lubricant. To prevent damage, the trachea is targeted using a small needle (Figure 1(b)). After aspiration of air and bronchoscopic confirmation of the correct position (midline, beneath second or third cartilage), an introducer needle is placed, and a guidewire is introduced. Next, an incision of approximately 1.5 cm is made, and after dilatation of the surrounding soft tissue (Figure 1(c)), the tracheostomy tube is placed in situ and secured (Figure 1(d)). The position control of the cannula is performed by bronchoscopy via cannula. PDT is performed under general anesthesia. The whole procedure is monitored by bronchoscopy under controlled ventilation with a FiO_2_ of 1.0. We do not routinely monitor for complications such as pneumothorax. In case of clinical suspicion, we do an ultrasound or chest X-ray.

In our ICU, we do platelet transfusion for patients with severe thrombocytopenia before starting PDT. The number of platelets transfused depends on the starting level of platelet counts and is based on experience to achieve values of >30 G/l; however, the actual value achieved by transfusion is not measured. In addition, no prophylactic antibiotics are administered.

### 2.2. The Procedure of OST

Patients with the need for tracheostomy who did not meet the indications for PDT were submitted to OST. This treatment was performed in the ear-nose-throat (ENT) department.

### 2.3. Definitions

Most of the patients were diagnosed with a hematooncological disease, and grading of conditions such as low platelet count and mucositis was according to the National Cancer Institute' common terminology criteria for adverse events (CTCAE). It is descriptive terminology that can be used for adverse events (AE) reporting [21].

#### 2.3.1. Platelet Count

Most patients experienced treatment-induced thrombocytopenia. Therefore, the platelet count at the time of the tracheostomy procedure was graded according to the CTCAE v5.0 criteria. According to these criteria, a decreased platelet count is defined as follows: grade 1 (lower limit of reference to 75 G/l), grade 2 (75—50 G/l), grade 3 (50—25 G/l), and grade 4 (<25 G/l).

#### 2.3.2. Mucositis

According to the CTCAE v5.0 criteria, mucositis is defined as follows: grade 1: asymptomatic or mild symptoms, intervention not indicated; grade 2: moderate pain or ulcer that does not interfere with oral intake, modified diet indicated; grade 3: severe pain, interfering with oral intake; grade 4: life-threatening consequences, urgent intervention indicated.

#### 2.3.3. Stoma Site Infection

A stoma site infection is a disorder characterized by an infectious process involving a stoma (surgically created opening of the surface of the body): grade 1: localized, local intervention indicated; grade 2: oral intervention indicated (e.g., antibiotic, antifungal, or antiviral); grade 3: IV antibiotic, antifungal, or antiviral intervention indicated, invasive intervention indicated; grade 4: life-threatening consequences, urgent intervention indicated.

#### 2.3.4. Coagulopathy

Coagulopathy/coagulation disorder was stated in case of therapeutic heparin administration, acute or acute-on-chronic liver failure (ACLF) with a quick value < 70%, vitamin K deficiency, or disseminated intravascular coagulation (DIC).

### 2.4. Statistical Analyses

Continuous normally distributed data were presented as means ± standard deviation (SD) and compared using Student's *t*-test. The Shapiro–Wilk test assessed normal distribution. Nonnormally distributed variables were presented by median with 1^st^ and 3^rd^ quartiles and compared using the Mann–Whitney *U* test. Correlation between data was examined using Pearson's correlation coefficient, chi-square test, or Fisher exact test. *P* values less than 0.05 were considered to indicate a statistical significance. All data were analyzed with SPSS version 26 (IBM Corp., Armonk, NY).

## 3. Results

Between May 2017 and November 2020, 63 patients underwent PDT, while 21 patients were submitted to OST during the same period. The leading causes of OST were severe obesity in eight cases, the need for permanent tracheostomy in five cases, blood vessels in the operating area in three cases, and a difficult airway in five cases.  Table 1 provides the baseline characteristics of both groups. Gender ratio, age, SAPS II, and ventilation time before tracheostomy were comparable between both groups; preexisting hematooncological diseases were not significantly increased in PDT patients. However, patients that underwent PDT were significantly more often after alloSCT (16 vs. 3 patients, *p*=0.033) and suffered from mucositis significantly more often (16 vs. 1 patient, *p*=0.043). The medical history of the patients is given in Table 2. There were no differences in leucocyte or platelet count on the tracheostomy day. Patients with coagulation disorders and patients under immunosuppression were distributed equally among both groups. Nearly all patients were under antibiotic therapy (95%). All eleven COVID-19 patients were treated with PDT.

Of the 63 patients, PDT was applied in the second intercartilaginous space for 27 patients (42.9%) and the third intercartilaginous space for the other 36 patients (57.1%). During the PDT procedure, fracture of tracheal cartilage happened in 13 cases (20.6%).

A stoma site infection was documented in five cases in the PDT group and eight cases in the OST group. In all cases, the grade of severity was low or moderate (CTCAE grades 1 and 2). CTCAE grade 2 infections were significantly increased in the OST group (0 vs. 3; *p*=0.002). Smears were positive in six cases in the PDT group; none of these patients had local infection signs. In the OST group, smears were positive in four cases, and all had signs of a stroma site infection. *Candida* and *Pseudomonas* species were the germs detected.

During PDT, periprocedural complications occurred in three cases (4.6%). In two cases, accidental extubation while pulling back the tube happened. The reintubation was successful without any decrease in saturation. One patient developed a spurting arterial hemorrhage after the dilation of the trachea. The bleeding stopped after insertion of the cannula. Periprocedural deaths were not observed, and no PDT procedure had to be aborted due to complications or technical challenges. There are no data on periprocedural complications during OST because the surgery reports do not give any information on this matter.

Postprocedural bleedings occurred in eight cases (9.5%) and significantly more often in the OST group (2 vs. 6 postprocedural bleedings, *p*=0.001), leading to emergency surgery in one case of the OST group. Six of them had a platelet count <25 G/l (PDT group 1 vs. OST group 5), and the other two had a normal platelet count.

In the PDT group, material deficiency was observed in one case. The cuff of the tracheal cannula was leaking; the cannula was replaced over a wire via the inserting bougie of the tracheostomy set the other day. Cardiopulmonary complications were observed in three cases, two cases of hypotension after anesthesia (3.1%) and one case of desaturation (1.5%).

Immunosuppressed patients suffered significantly more often from mucositis than immunocompetent patients (16 vs. 1 patient (26.7 vs. 4.2%); *p*=0.002). There was no significant correlation found between the way of tracheostomy (*p*=0.595), immunosuppression (*p*=1.0), antibiotic therapy (*p*=0.126), mucositis (*p*=1.0), hematooncological diseases (*p*=0.659), alloSCT (*p*=1.0), transplantation (*p*=1.0), and COVID-19 (*p*=0.513) and local site infections. Furthermore, no correlation was found between preexisting immunosuppression and fractures of cartilage (*p*=0.434), as well as there was no correlation between thrombocytopenia (Χ^2^ 67.559; *p*=0.626), coagulation disorders (*p*=0.683), and postprocedural bleeding complications.

## 4. Discussion

Tracheostomies are standard procedures in ICU. PDT and OST are the techniques of choice and clinical practice [7]. So far, data on tracheostomies in patients under immunosuppression are scarce [11]. This study aimed to examine the feasibility of PDT in an immunosuppressed and thrombocytopenic patient cohort.

Eighty-four patients needing tracheostomy were included; 63 patients received PDT and 21 OST. Both groups were comparable regarding baseline characteristics such as sex, age, SAPS II, ventilations days before tracheostomy, and preexisting morbidities. Most of the patients were under immunosuppression (71.4%) and suffered from thrombocytopenia (57.1%), indicating that the study cohort was representative to evaluate the feasibility of PDT in immunosuppressed and thrombocytopenic patients.

Complications in the overall cohort were rare (15.5% stoma site infection and 9.5% postprocedural bleedings) and in line with the complication rate reported by other studies and reviews [2, 18]. The severity grade of the stoma site infections was low to moderate (CTCAE grades 1 and 2), and no higher-grade stoma site infections occurred. Although the patients of the PDT group were significantly more often after alloSCT (PDT 25.4% vs. OST 14.3%, *p*=0.033) and had a significantly higher rate of mucositis (PDT 23.8% vs. OST 4.8%), the cases of stoma site infections were smaller, even if it was not significant (PDT 7.9% vs. OST 38.1%, *p*=0.427). Similar results were reported by Botti et al. in a COVID-19 patient cohort [22]. One reason for the higher rate of infections in the OST group might be the humid milieu of the wound. Many patients needing tracheostomy after critical illness are suffering from swallowing dysfunction [23]. This leads to constant lacking saliva out of the stoma. Due to the humidity and saliva germs, the risk of infection is increased. PDT leads to a close fitting of the tracheal cannula to the stoma; humid wound conditions are sporadic. Suzuki et al. concluded in their study that the small skin incision during the PDT procedure leads to fewer postoperative complications and is, therefore, a safer procedure than OST to be performed in ICU [24].

Germs detected in smears of the stoma site were *Candida* and *Pseudomonas* species, which are normal germs of the dermal flora. Only four patients of the OST group with positive smears had local infection signs simultaneously. Prophylactic antibiotics are not administered in our ICU in the perioperative setting of tracheostomy as the incidence of bacteremia following PDT was similar to other manipulations of the aerodigestive tract such as intubation or tooth brushing [12]. Nevertheless, nearly all patients (95%) were under antibiotic treatment due to other causes. This may explain the low rate of stoma site infections in the overall cohort.

Thrombocytopenia was present in both groups and equally distributed over severity grades (CTCAE grades 1–4). A small proportion suffered from coagulation disorders (PDT 15.9% vs. OST 19.0%). One relevant periprocedural bleeding (spurting arterial hemorrhage) occurred during PDT but could be stopped by inserting the cannula without further bleeding or harming the patient. Minor bleedings during PDT after incision were not included in this analysis. Data on periprocedural bleedings of the OST group are not available as the surgery reports are not giving any information on this matter. Only one reported relevant periprocedural bleeding out of 63 patients in the PDT group is very small and smaller than reported bleeding complications [18]. Postprocedural bleeding complications were significantly increased in the OST group (PDT 3.2% vs. OST 28.6%; *p*=0.001). This result is surprising as the rate of thrombocytopenia and coagulation disorders was equally distributed in both groups, and due to the surgical approach, hemostasis could be performed more subtle during OST.

In two patients in the PDT group, accidental extubation while pulling back the tube occurred, the reintubation was successful without any decrease in saturation. This complication is unique for the procedure of PDT as the tube during OST is usually pushed forward towards the main carina to avoid damage to the cuff during skin incision. As an uncomplicated airway with a low Cormack-Lehane grade is required for PDT [25], complications such as accidental extubation should never lead to a life-threatening situation.

Fracture of the cartilage is another unique complication of PDT and happened in 13 cases. There was no correlation between this complication and the immunosuppressive therapy and has, therefore, in our view, to be considered independent of it.

All COVID-19 patients were treated with PDT; none of the medical team was infected due to the procedure. The rate of complications was similar to other patient groups, and PDT can therefore be considered safe for COVID-19 patients, which is in line with the results of other studies [26–28].

Our study has some limitations. First, the cohort is small, and the data represent a single-center experience and are recorded retrospectively. Prospective, multicenter studies with larger patient cohorts are needed to confirm these results. Second, nearly all patients were under antibiotic treatment, which might have reduced the risk for infections. On the other site, no prophylactic antibiotics were administered; all treatments were due to other infections and could therefore not be stopped.

## 5. Conclusion

PDT is a feasible and safe procedure in a predominantly immunosuppressed and thrombocytopenic patient cohort without an increased risk for stoma site infections or bleeding complications.

## Figures and Tables

**Table 1:** Baseline characteristics and data from ICU.

Baseline characteristics and data from ICU				
Parameter	All patients	PDT patients	OST patients	*P* value
Patients, *n* (%)	84 (100)	63 (75)	21 (25)	
Female, *n* (%) per group	35 (41.7)	25 (39.7)	10 (47.6)	0.613
Age (y)	57.5 (49.3–67.8)	60 (51–68)	53 (43–63.5)	0.070
SAPS II	45.3 ± 13.6	47.4 ± 13.4	39.7 ± 10.9	0.084
Ventilation before PDT/OST (d)	13 (9–17)	13 (9–17)	12 (7–17)	0.472
Hematological/oncological disease, *n* (%)	46 (54.7)	39 (61.9)	7 (33.3)	0.156
AlloSCT, *n* (%)	19 (22.6)	16 (25.4)	3 (14.3)	0.033
Mucositis, *n* (%)	17 (20.2)	16 (25.4)	1 (4.8)	0.043
CTCAE grade 1, *n* (%)	7 (8.3)	7 (11.1)	0	0.133
CTCAE grade 2, *n* (%)	5 (6.0)	5 (7.9)	0	0.186
CTCAE grade 3, *n* (%)	5 (6.0)	4 (6.3)	1 (4.8)	0.791
CTCAE grade 4, *n* (%)	0	0	0	—
Solid organ transplant, *n* (%)	8 (9.5)	4 (6.3)	4 (19.0)	0.088
COVID-19, *n* (%)	11 (13.1)	11 (17.5)	0 (0)	0.041
Leucocyte counts (G/l)	9.88 (4.26–15.65)	8.58 (3.3.8–14.1)	11.5 (5.96–16.4)	0.273
Platelet counts (G/l)	92.5 (29–201)	86 (28–204)	125 (44–1195)	0.602
CTCAE grade 1, *n* (%)	13 (15.5)	9 (14.3)	4 (19.0)	0.524
CTCAE grade 2, *n* (%)	8 (9.5)	6 (9.5)	2 (9.5)	1.000
CTCAE grade 3, *n* (%)	12 (14.3)	10 (15.9)	2 (9.5)	0.699
CTCAE grade 4, *n* (%)	15 (17.9)	11 (17.5)	4 (19.0)	0.876
Anticoagulation with heparin, *n* (%)	17 (20.2)	13 (20.6)	4 (19.0)	0.876
Coagulopathy (ACLF), *n* (%)	7 (8.3)	7 (11.1)	0 (0)	0.113
Immunosuppression, *n* (%)	60 (71.4)	49 (77.8)	11 (52.4)	0.302
Antibiotic therapy, *n* (%)	80 (95)	60 (95.2)	20 (95.2)	0.095
Complications				
Fracture of cartilage, *n* (%)		13 (20.6)	—	
Stoma site infection, *n* (%)	13 (15.5)	5 (7.9)	8 (38.1)	0.427
CTCAE grade 1, *n* (%)	10 (11.9)	5 (7.9)	5 (23.8)	0.053
CTCAE grade 2, *n* (%)	3 (3.6)	0	3 (14.3)	0.002
CTCAE grade 3, *n* (%)	0	0	0	—
CTCAE grade 4, *n* (%)	0	0	0	—
Smears positive, *n* (%)	10 (11.9)	6 (9.5)	4 (19.0)	0.156
Bleeding postprocedural, *n* (%)	8 (9.5)	2 (3.2)	6 (28.6)	0.001
Length of stay in ICU (d)	32.5 (24–53)	34 (25–53)	31 (19–54)	0.423

The data are mean values ± standard deviation, median with interquartile range, or number of patients, and in brackets, the percentage of the respective group.

**Table 2:** Medical history of all patients; patients can be counted more than once due to multimorbidity.

Medical history		
Tracheostomy	PDT	OST
Patients	63	21
Immunosuppression	49 (77.8)	11 (52.4)
Hematological/oncological disease	39 (61.9)	7 (33.3)
Acute leukemia	15 (23.8)	4 (19)
Acute myeloid leukemia	10 (15.9)	3 (14.3)
Acute lymphatic leukemia	4 (6.3)	1 (4.8)
Lymphoma	15 (23.8)	1 (4.8)
Non-Hodgkin's lymphoma	13 (20.6)	1 (4.8)
Hodgkin's lymphoma	1 (1.6)	—
PTLD after lung transplant	1 (1.6)	—
Natural killer cell leukemia	1 (1.6)	—
Myelodysplastic syndrome	4 (6.3)	1 (4.8)
Multiple myeloma	4 (6.3)	1 (4.8)
Solid tumor	3 (4.8)	1 (4.8)
lung transplantation	2 (3.2)	4 (19)
COPD	8 (12.7)	3 (14.3)
Mucoviscidosis	—	2 (9.5)
ARDS	2 (3.2)	—
Silicosis	1 (1.6)	—
Lung fibrosis	—	1 (4.8)
Pneumothorax	1 (1.6)	—
Hepatopathy	1 (1.6)	—
Cirrhosis of the liver	3 (4.8)	2 (9.5)
Necrotizing pancreatitis	1 (1.6)	—
Mechanical ileus	1 (1.6)	—
Retention stomach	1 (1.6)	—
Gastrointestinal bleeding	1 (1.6)	—
Obesity	1 (1.6)	8 (38.1)
Diabetes mellitus	4 (6.3)	—
Kidney transplantation	2 (3.2)	—
Meningococcal sepsis	—	1 (4.8)
Allogeneic stem cell transplantation	16 (25.4)	3 (14.3)
Chimeric antigen receptor T cell therapy	1 (1.6)	—

The percentage refers to the total number of patients per group, so that multimorbidity can lead to more than 100%.

## Data Availability

The data used to support the results of this study are included within the article.
